# Supplementary material for: Machine Learning Accelerates Crystallization for Structure Determination
Source: Angew Chem Int Ed Engl. 2026 May 4;65(25):e1218503. doi: 10.1002/anie.1218503 (PMC13266958; doi:10.1002/anie.1218503)

checkCIF/PLATON report

Structure factors have been supplied for datablock(s) lcz040

THIS REPORT IS FOR GUIDANCE ONLY. IF USED AS PART OF A REVIEW PROCEDURE FOR PUBLICATION, IT SHOULD NOT REPLACE THE EXPERTISE OF AN EXPERIENCED CRYSTALLOGRAPHIC REFEREE.

No syntax errors found. CIF dictionary Interpreting this report

Datablock: lcz040

Bond precision: C-C = 0.0171 A Wavelength=1.54184

Cell:	a=15.2112(2)	b=17.7741(3)	c=22.4082(3)
	alpha=90.086(1)	beta=91.166(1)	gamma=113.565(2)
Temperature:	100 K		

	Calculated	Reported
Volume	5551.72(16)	5551.71(16)
Space group	P -1	P -1
Hall group	-P 1	-P 1
Moiety formula	C46 H20 Ag6 F36 N12 O2, C47 H31 Ag3 F18 N6 O4	C15 H3 Ag3 F18 N6, C16 H14 O2
Sum formula	C93 H51 Ag9 F54 N18 O6	C31 H17 Ag3 F18 N6 O2
Mr	3513.35	1171.11
Dx,g cm-3	2.102	2.102
Z	2	6
Mu (mm-1)	13.834	13.834
F000	3384.0	3384.0
F000’	3399.91	
h,k,lmax	18,21,26	18,21,26
Nref	19605	19453
Tmin,Tmax	0.069,0.036	0.622,1.000
Tmin’	0.011	

Correction method= # Reported T Limits: Tmin=0.622 Tmax=1.000
AbsCorr = MULTI-SCAN

Data completeness= 0.992 Theta(max)= 66.599

R(reflections)= 0.0693(16564) wR2(reflections)= 0.1881(19453)

S = 1.029 Npar= 1594

The following ALERTS were generated. Each ALERT has the format
test-name_ALERT_alert-type_alert-level.
Click on the hyperlinks for more details of the test.

Alert level A

PLAT971_ALERT_2_A Check Calcd Resid. Dens. 0.99Ang From N00U 3.90 eA-3

Author Response: This Alert is due to presence of residual density in the presence of heavy metal atom (Ag).

PLAT971_ALERT_2_A Check Calcd Resid. Dens. 1.21Ang From N012 3.88 eA-3

Author Response: This Alert is due to presence of residual density in the presence of heavy metal atom (Ag).

Alert level B

PLAT971_ALERT_2_B Check Calcd Resid. Dens. 1.49Ang From C04P 2.93 eA-3

Author Response: This Alert is due to presence of residual density in the presence of heavy metal atom (Ag).

PLAT971_ALERT_2_B Check Calcd Resid. Dens. 1.08Ang From C2 2.65 eA-3

Author Response: This Alert is due to presence of residual density in the presence of heavy metal atom (Ag).

PLAT971_ALERT_2_B Check Calcd Resid. Dens. 1.06Ang From C04X 2.63 eA-3

Author Response: This Alert is due to presence of residual density in the presence of heavy metal atom (Ag).

PLAT971_ALERT_2_B Check Calcd Resid. Dens. 1.10Ang From C04T 2.56 eA-3

Author Response: This Alert is due to presence of residual density in the presence of heavy metal atom (Ag).

Alert level C

```
DIFMX02_ALERT_1_C  The maximum difference density is > 0.1*ZMAX*0.75
            The relevant atom site should be identified.
PLAT041_ALERT_1_C Calc. and Reported SumFormula    Strings  Differ     Please Check
              Calc: C93 H51 Ag9 F54 N18 O6
              Rep.: C31 H17 Ag3 F18 N6 O2
PLAT042_ALERT_1_C Calc. and Reported MoietyFormula Strings  Differ     Please Check
              Calc: C46 H20 Ag6 F36 N12 O2, C47 H31 Ag3 F18 N6 O4
              Rep.: C15 H3 Ag3 F18 N6, C16 H14 O2
PLAT094_ALERT_2_C Ratio of Maximum / Minimum Residual Density ....       3.15 Report
PLAT097_ALERT_2_C Large Reported Max.  (Positive) Residual Density       3.84 eA-3
PLAT213_ALERT_2_C Atom F02T            has ADP max/min Ratio .....        3.1 prolat
PLAT213_ALERT_2_C Atom F03W            has ADP max/min Ratio .....        3.1 prolat
PLAT213_ALERT_2_C Atom N00C            has ADP max/min Ratio .....        3.5 prolat
PLAT220_ALERT_2_C NonSolvent   Resd 1  F   Ueq(max)/Ueq(min) Range        3.5 Ratio
PLAT230_ALERT_2_C Hirshfeld Test Diff for    O03P     --C2        .       6.6 s.u.
PLAT230_ALERT_2_C Hirshfeld Test Diff for    C037     --C04M      .       5.6 s.u.
PLAT230_ALERT_2_C Hirshfeld Test Diff for    C038     --C03U      .       6.0 s.u.
PLAT234_ALERT_4_C Large Hirshfeld Difference F02S     --C03O      .      0.20 Ang.
PLAT234_ALERT_4_C Large Hirshfeld Difference F04K     --C040      .      0.17 Ang.
PLAT234_ALERT_4_C Large Hirshfeld Difference O00O     --C03C      .      0.19 Ang.
PLAT234_ALERT_4_C Large Hirshfeld Difference O00R     --C03X      .      0.16 Ang.
PLAT234_ALERT_4_C Large Hirshfeld Difference O031     --C04M      .      0.16 Ang.
PLAT234_ALERT_4_C Large Hirshfeld Difference O03P     --C04T      .      0.23 Ang.
PLAT234_ALERT_4_C Large Hirshfeld Difference C2       --C03I      .      0.21 Ang.
PLAT234_ALERT_4_C Large Hirshfeld Difference C4       --C03X      .      0.16 Ang.
PLAT234_ALERT_4_C Large Hirshfeld Difference C02F     --C048      .      0.18 Ang.
PLAT234_ALERT_4_C Large Hirshfeld Difference C03N     --C04O      .      0.22 Ang.
PLAT234_ALERT_4_C Large Hirshfeld Difference C03V     --C04X      .      0.17 Ang.
PLAT234_ALERT_4_C Large Hirshfeld Difference C04X     --C02I      .      0.17 Ang.
PLAT234_ALERT_4_C Large Hirshfeld Difference C054     --C02I      .      0.17 Ang.
PLAT241_ALERT_2_C High   'MainMol' Ueq as Compared to Neighbors of      O03P Check
PLAT241_ALERT_2_C High   'MainMol' Ueq as Compared to Neighbors of        C5 Check
PLAT241_ALERT_2_C High   'MainMol' Ueq as Compared to Neighbors of      C044 Check
PLAT241_ALERT_2_C High   'MainMol' Ueq as Compared to Neighbors of      C048 Check
PLAT241_ALERT_2_C High   'MainMol' Ueq as Compared to Neighbors of      C04O Check
PLAT241_ALERT_2_C High   'MainMol' Ueq as Compared to Neighbors of      C04P Check
PLAT242_ALERT_2_C Low    'MainMol' Ueq as Compared to Neighbors of      C037 Check
PLAT242_ALERT_2_C Low    'MainMol' Ueq as Compared to Neighbors of      C03I Check
PLAT242_ALERT_2_C Low    'MainMol' Ueq as Compared to Neighbors of      C02I Check
PLAT332_ALERT_2_C Large Phenyl C-C Range     C03N      -C04O      .      0.19 Ang.
PLAT342_ALERT_3_C Low Bond Precision on  C-C Bonds ...............    0.01711 Ang.
PLAT369_ALERT_2_C Long   C(sp2)-C(sp2) Bond  C4        - C03X      .      1.53 Ang.
PLAT369_ALERT_2_C Long   C(sp2)-C(sp2) Bond  C02F      - C03M      .      1.54 Ang.
PLAT413_ALERT_2_C Short Inter XH3 .. XHn     H04N     ..H05A      .      2.09 Ang.
                                               1-x,2-y,1-z  =      2_676 Check
PLAT906_ALERT_3_C Large K Value in the Analysis of Variance ......      5.075 Check
PLAT911_ALERT_3_C Missing FCF Refl Between Thmin & STh/L=    0.595        151 Report
               16  1  0, -17  2  0,  15  3  0,  10-20  1,   8-18  1,  17 -3  1,
               17 -2  1, -16  0  1,  16  0  1,  15  1  1,  16  1  1,  15  2  1,
                7 14  1,   6 15  1,   4 17  1,   6-10  2,  17 -4  2,  17 -3  2,
               17 -2  2,  16 -1  2,  16  0  2,  15  1  2,  16  1  2,  14  2  2,
               15  3  2,  -1  4  2,  14  5  2,  -3-17  3, -12 -8  3,   5 -8  3,
                   (  121 More Missing: see the .ckf listing file)
PLAT971_ALERT_2_C Check Calcd Resid. Dens.  0.97Ang From C4             2.48 eA-3
```

Author Response: This Alert is due to presence of residual density in the presence of heavy metal atom (Ag).

PLAT971_ALERT_2_C Check Calcd Resid. Dens. 1.04Ang From C03V 2.42 eA-3

Author Response: This Alert is due to presence of residual density in the presence of heavy metal atom (Ag).

PLAT971_ALERT_2_C Check Calcd Resid. Dens. 0.81Ang From C04N 2.12 eA-3

Author Response: This Alert is due to presence of residual density in the presence of heavy metal atom (Ag).

PLAT971_ALERT_2_C Check Calcd Resid. Dens. 0.90Ang From Ag05 2.09 eA-3

Author Response: This Alert is due to presence of residual density in the presence of heavy metal atom (Ag).

PLAT971_ALERT_2_C Check Calcd Resid. Dens. 0.82Ang From C053 2.03 eA-3

Author Response: This Alert is due to presence of residual density in the presence of heavy metal atom (Ag).

PLAT971_ALERT_2_C Check Calcd Resid. Dens. 0.92Ang From Ag04 2.02 eA-3

Author Response: This Alert is due to presence of residual density in the presence of heavy metal atom (Ag).

PLAT971_ALERT_2_C Check Calcd Resid. Dens. 0.29Ang From C4 2.00 eA-3

Author Response: This Alert is due to presence of residual density in the presence of heavy metal atom (Ag).

PLAT971_ALERT_2_C Check Calcd Resid. Dens. 0.88Ang From Ag04 1.97 eA-3

Author Response: This Alert is due to presence of residual density in the presence of heavy metal atom (Ag).

PLAT971_ALERT_2_C Check Calcd Resid. Dens. 0.84Ang From Ag08 1.96 eA-3

Author Response: This Alert is due to presence of residual density in the presence of heavy metal atom (Ag).

PLAT971_ALERT_2_C Check Calcd Resid. Dens. 0.95Ang From Ag08 1.96 eA-3

Author Response: This Alert is due to presence of residual density in the presence of heavy metal atom (Ag).

PLAT971_ALERT_2_C Check Calcd Resid. Dens. 1.01Ang From Ag01 1.94 eA-3

Author Response: This Alert is due to presence of residual density in the presence of heavy metal atom (Ag).

PLAT971_ALERT_2_C Check Calcd Resid. Dens. 0.60Ang From C1 1.94 eA-3

Author Response: This Alert is due to presence of residual density in the presence of heavy metal atom (Ag).

PLAT971_ALERT_2_C Check Calcd Resid. Dens. 1.04Ang From Ag07 1.86 eA-3

Author Response: This Alert is due to presence of residual density in the presence of heavy metal atom (Ag).

PLAT971_ALERT_2_C Check Calcd Resid. Dens. 0.99Ang From Ag06 1.86 eA-3

Author Response: This Alert is due to presence of residual density in the presence of heavy metal atom (Ag).

PLAT971_ALERT_2_C Check Calcd Resid. Dens. 1.00Ang From Ag01 1.79 eA-3

Author Response: This Alert is due to presence of residual density in the presence of heavy metal atom (Ag).

PLAT971_ALERT_2_C Check Calcd Resid. Dens. 1.16Ang From Ag02 1.79 eA-3

Author Response: This Alert is due to presence of residual density in the presence of heavy metal atom (Ag).

PLAT971_ALERT_2_C Check Calcd Resid. Dens. 0.95Ang From Ag03 1.76 eA-3

Author Response: This Alert is due to presence of residual density in the presence of heavy metal atom (Ag).

PLAT971_ALERT_2_C Check Calcd Resid. Dens. 1.13Ang From Ag05 1.74 eA-3

Author Response: This Alert is due to presence of residual density in the presence of heavy metal atom (Ag).

```
PLAT971_ALERT_2_C Check Calcd Resid. Dens.  0.57Ang From N00C           1.73 eA-3
```

Author Response: This Alert is due to presence of residual density in the presence of heavy metal atom (Ag).

```
PLAT977_ALERT_2_C Check Negative Difference Density on H03Q      .      -0.39 eA-3
```

Alert level G

```
PLAT002_ALERT_2_G Number of Distance or Angle Restraints on AtSite          9 Note
PLAT003_ALERT_2_G Number of Uiso or U(i,j)  Restrained non-H-Atoms         30 Report
PLAT045_ALERT_1_G Calculated and Reported Z Differ by a Factor ...      0.333 Check
PLAT083_ALERT_2_G SHELXL Second Parameter in WGHT  Unusually Large      53.10 Why ?
PLAT171_ALERT_4_G The CIF-Embedded .res File Contains EADP Records          1 Report
PLAT172_ALERT_4_G The CIF-Embedded .res File Contains DFIX Records          5 Report
PLAT177_ALERT_4_G The CIF-Embedded .res File Contains DELU Records         14 Report
PLAT178_ALERT_4_G The CIF-Embedded .res File Contains SIMU Records          7 Report
PLAT186_ALERT_4_G The CIF-Embedded .res File Contains ISOR Records          6 Report
PLAT188_ALERT_3_G A Non-default SIMU Restraint Value has been used     0.0100 Report
PLAT188_ALERT_3_G A Non-default SIMU Restraint Value has been used     0.0100 Report
PLAT188_ALERT_3_G A Non-default SIMU Restraint Value has been used     0.0100 Report
PLAT192_ALERT_3_G A Non-default DELU Restraint Value for SecondPar     0.0200 Report
PLAT192_ALERT_3_G A Non-default DELU Restraint Value for SecondPar     0.0200 Report
PLAT192_ALERT_3_G A Non-default DELU Restraint Value for SecondPar     0.0200 Report
PLAT192_ALERT_3_G A Non-default DELU Restraint Value for SecondPar     0.0200 Report
PLAT192_ALERT_3_G A Non-default DELU Restraint Value for SecondPar     0.0200 Report
PLAT192_ALERT_3_G A Non-default DELU Restraint Value for SecondPar     0.0200 Report
PLAT232_ALERT_2_G Hirshfeld Test Diff (M-X)  Ag02     --N00C     .        6.0 s.u.
PLAT242_ALERT_2_G Low    'MainMol' Ueq as Compared to Neighbors of       C024 Check
PLAT242_ALERT_2_G Low    'MainMol' Ueq as Compared to Neighbors of       C02V Check
PLAT242_ALERT_2_G Low    'MainMol' Ueq as Compared to Neighbors of       C032 Check
PLAT242_ALERT_2_G Low    'MainMol' Ueq as Compared to Neighbors of       C036 Check
PLAT242_ALERT_2_G Low    'MainMol' Ueq as Compared to Neighbors of       C039 Check
PLAT242_ALERT_2_G Low    'MainMol' Ueq as Compared to Neighbors of       C03A Check
PLAT242_ALERT_2_G Low    'MainMol' Ueq as Compared to Neighbors of       C03H Check
PLAT242_ALERT_2_G Low    'MainMol' Ueq as Compared to Neighbors of       C03O Check
PLAT242_ALERT_2_G Low    'MainMol' Ueq as Compared to Neighbors of       C03R Check
PLAT242_ALERT_2_G Low    'MainMol' Ueq as Compared to Neighbors of       C040 Check
PLAT242_ALERT_2_G Low    'MainMol' Ueq as Compared to Neighbors of       C041 Check
PLAT242_ALERT_2_G Low    'MainMol' Ueq as Compared to Neighbors of       C043 Check
PLAT242_ALERT_2_G Low    'MainMol' Ueq as Compared to Neighbors of       C03F Check
PLAT242_ALERT_2_G Low    'MainMol' Ueq as Compared to Neighbors of       C04A Check
PLAT242_ALERT_2_G Low    'MainMol' Ueq as Compared to Neighbors of       C04E Check
PLAT242_ALERT_2_G Low    'MainMol' Ueq as Compared to Neighbors of       C04G Check
PLAT242_ALERT_2_G Low    'MainMol' Ueq as Compared to Neighbors of       C04H Check
PLAT242_ALERT_2_G Low    'MainMol' Ueq as Compared to Neighbors of       C04R Check
PLAT720_ALERT_4_G Number of Unusual/Non-Standard Labels ..........        223 Note
              Ag01    Ag02    Ag03    Ag04    Ag05    Ag06    Ag07    Ag08
              Ag09    F00A    F00B    N00C    F00D    F00E    F00F    F00G
              O00H    F00I    F00J    F00K    F00L    F00M    F00N    O00O
              F00P    F00Q    O00R    N00S    F00T    N00U    F00V    F00W
```

```
              F00X    O00Y    F00Z    F010    F011    N012    F013    N014
              N015    F016    N017    F018    N019    F01A    N01B    N01C
              N01D    N01E    N01F    F01G    N01H    F01I    N01J    F01K
              N01L    F01M    F01N    F01O    F01P    F01Q    F01R    N01S
              F01T    N01U    F01V    F01W    F01X    F01Y    C01Z    F020
              C021    C022    H022    F023    C024    F025    C026    H026
              C027    C028    H028    F029    C02A    C02B    C02C    C02D
              H02D    C02E    H02E    C02F    C02G    C02H    H02H    C02J
              C02K    H02K    C02L    C02M    C02N    H02N    C02O    H02O
              C02P    H02P    F02Q    C02R    F02S    F02T    C02U    C02V
              C02W    F02X    C02Y    F02Z    C030    O031    C032    C033
              H033    C034    C035    H035    C036    C037    H037    C038
              H038    C039    C03A    C03B    C03C    H03A    H03B    H03C
              C03D    C03E    C03F    F03G    C03H    C03I    H03I    C03J
              H03J    C03K    C03M    C03N    H03N    C03O    O03P    C03Q
              H03Q    C03R    C03S    H03S    C03T    H03T    C03U    H03U
              C03V    H03V    F03W    C03X    C03Y    H03Y    C03Z    C040
              C041    C042    H042    C043    C044    H044    F045    C046
              H046    F047    C048    H048    C049    H049    C04A    C04B
              C04C    C04D    H04D    C04E    C04F    H04F    C04G    C04H
              C04I    H04I    F04J    F04K    C04M    C04O    H04O    C04P
              H04P    C04R    C04S    H04S    C04T    H04A    H04B    H04C
              C04X    C053    H053    C04N    H04N    C04U    H04U    C054
              H054    C02I    H02I    C050    H05A    H05B    H05C
PLAT860_ALERT_3_G Number of Least-Squares Restraints ............        238 Note
PLAT883_ALERT_1_G Absent Datum for _atom_sites_solution_primary ..     Please Do !
PLAT909_ALERT_3_G Percentage of I>2sig(I) Data at Theta(Max) Still       76% Note
PLAT910_ALERT_3_G Missing FCF Reflection(s) Below Theta(Min)[Deg]=      2.71 Note
                0  0  1,
PLAT933_ALERT_2_G Number of HKL-OMIT Records in Embedded .res File        30 Note
              -17  0  3, -17  0  4, -17  1  2, -16 -2  1, -16 -2  2, -16  0  1,
              -15 -4  3, -12 -8  3,  13  7  3,  13  8  1,  13  8  3,  14 -1  5,
               14  2  2,  14  5  2,  14  5  4,  14  5  6,  14  6  2,  15  0  3,
               15  1  1,  15  1  2,  15  1  3,  15  2  1,  15  2  3,  15  3  0,
               15  3  2,  15  3  3,  15  4  1,  15  4  2,  16  0  1,  16  1  0,
PLAT941_ALERT_3_G Average HKL Measurement Multiplicity ..........        3.4 Low
PLAT969_ALERT_5_G The 'Henn et al.' R-Factor-gap value ..........      3.749 Note
              Predicted wR2: Based on SigI**2  5.02 or SHELX Weight 18.28
PLAT978_ALERT_2_G Number C-C Bonds with Positive Residual Density.          1 Info
```

---

2 **ALERT level A** = Most likely a serious problem - resolve or explain
4 **ALERT level B** = A potentially serious problem, consider carefully
61 **ALERT level C** = Check. Ensure it is not caused by an omission or oversight
46 **ALERT level G** = General information/check it is not something unexpected

5 ALERT type 1 CIF construction/syntax error, inconsistent or missing data
72 ALERT type 2 Indicator that the structure model may be wrong or deficient
16 ALERT type 3 Indicator that the structure quality may be low
19 ALERT type 4 Improvement, methodology, query or suggestion
1 ALERT type 5 Informative message, check

It is advisable to attempt to resolve as many as possible of the alerts in all categories. Often the minor alerts point to easily fixed oversights, errors and omissions in your CIF or refinement strategy, so attention to these fine details can be worthwhile. It is up to the individual to critically assess their own results and, if necessary, seek expert advice.

Validation response form

Please find below a validation response form (VRF) that can be filled in and pasted into your CIF.

```
# start Validation Reply Form
_vrf_DIFMX02_lcz040
;
PROBLEM: The maximum difference density is > 0.1*ZMAX*0.75
RESPONSE: ...
;
_vrf_PLAT041_lcz040
;
PROBLEM: Calc. and Reported SumFormula    Strings  Differ     Please Check
RESPONSE: ...
;
_vrf_PLAT042_lcz040
;
PROBLEM: Calc. and Reported MoietyFormula Strings  Differ     Please Check
RESPONSE: ...
;
_vrf_PLAT094_lcz040
;
PROBLEM: Ratio of Maximum / Minimum Residual Density ....       3.15 Report
RESPONSE: ...
;
_vrf_PLAT097_lcz040
;
PROBLEM: Large Reported Max.  (Positive) Residual Density       3.84 eA-3
RESPONSE: ...
;
_vrf_PLAT213_lcz040
;
PROBLEM: Atom F02T            has ADP max/min Ratio .....        3.1 prolat
RESPONSE: ...
;
_vrf_PLAT220_lcz040
;
PROBLEM: NonSolvent   Resd 1  F   Ueq(max)/Ueq(min) Range        3.5 Ratio
RESPONSE: ...
;
_vrf_PLAT230_lcz040
;
PROBLEM: Hirshfeld Test Diff for    O03P     --C2       .        6.6 s.u.
RESPONSE: ...
;
_vrf_PLAT234_lcz040
;
PROBLEM: Large Hirshfeld Difference F02S     --C03O     .       0.20 Ang.
RESPONSE: ...
;
```

```
_vrf_PLAT241_lcz040
;
PROBLEM: High   'MainMol' Ueq as Compared to Neighbors of       O03P Check
RESPONSE: ...
;
_vrf_PLAT242_lcz040
;
PROBLEM: Low    'MainMol' Ueq as Compared to Neighbors of       C037 Check
RESPONSE: ...
;
_vrf_PLAT332_lcz040
;
PROBLEM: Large Phenyl C-C Range     C03N      -C04O     .       0.19 Ang.
RESPONSE: ...
;
_vrf_PLAT342_lcz040
;
PROBLEM: Low Bond Precision on  C-C Bonds ...............    0.01711 Ang.
RESPONSE: ...
;
_vrf_PLAT369_lcz040
;
PROBLEM: Long   C(sp2)-C(sp2) Bond  C4       - C03X     .       1.53 Ang.
RESPONSE: ...
;
_vrf_PLAT413_lcz040
;
PROBLEM: Short Inter XH3 .. XHn     H04N     ..H05A     .       2.09 Ang.
RESPONSE: ...
;
_vrf_PLAT906_lcz040
;
PROBLEM: Large K Value in the Analysis of Variance ......      5.075 Check
RESPONSE: ...
;
_vrf_PLAT911_lcz040
;
PROBLEM: Missing FCF Refl Between Thmin & STh/L=    0.595        151 Report
RESPONSE: ...
;
_vrf_PLAT977_lcz040
;
PROBLEM: Check Negative Difference Density on H03Q      .      -0.39 eA-3
RESPONSE: ...
;
# end Validation Reply Form
```

PLATON version of 17/09/2025; check.def file version of 17/09/2025

---

duplicate check

No duplication found

Datablock lcz040 - ellipsoid plot

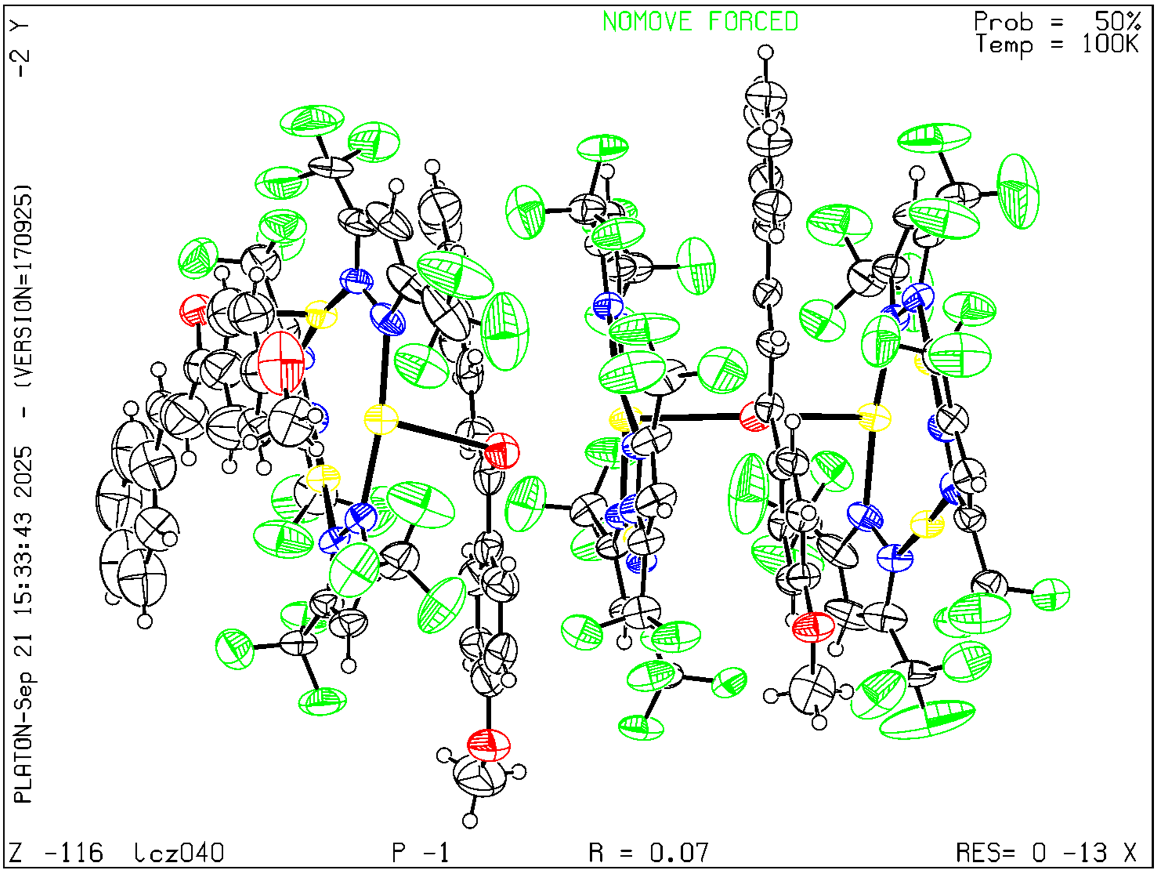

Supplement: Supplementary file 2 — Supporting File 2: anie72462‐sup‐0002‐DataFile.zip. [file ANIE-65-e1218503-s002.zip › supplementary_materials_crystallographic_data submit.zip!/cif_and_checkcif(1-114)/Ag3Pz3_40/Ag3Pz3_40_checkcif.pdf]
